# Intraoperative blood loss, postoperative drainage, and recovery in patients undergoing lumbar spinal surgery

**DOI:** 10.1186/s12893-015-0062-9

**Published:** 2015-06-20

**Authors:** Haibo Zou, Zhongshi Li, Houfu Sheng, Mingsheng Tan, Feng Yang, Li Liang, Jingxin Zhao

**Affiliations:** Department of Orthopaedic Spine Surgery, China-Japan Friendship Hospital, 2 Yinghua Dongjie, Hepingli, Chaoyang District, Beijing, 100029 P. R. China

**Keywords:** Intraoperative blood loss, Postoperative drainage volume, Close and open of drainage tube, Spine surgery

## Abstract

**Background:**

Spine surgery is widely accepted as an effective management for patients with lumbar disc herniation; however, the factors influencing intraoperative procedure and prognosis are not fully understood. The present study was aimed to identify the factors influencing intraoperative blood loss, postoperative drainage volume, and recovery in patients undergoing spinal surgery.

**Methods:**

We retrospectively analyzed the clinical data of 183 consecutive patients with lumbar disc herniation who underwent spine surgery. The clinical characteristics, operation procedure, and outcome were documented and the correlations were analyzed.

**Results:**

There were significant differences between one-level and two-level operations in the bleeding volumes of male (*P* = 0.005) and female (*P* = 0.002) patients, and in final drainage of male (*P* = 0.043) and female (*P* = 0.003) patients. The blood loss was correlated with the operation duration. There were differences in intraoperative bleeding and final drainage between groups with one-level and two-level operations. Additionally, there were differences in intraoperative autologous blood transfusion among various groups. There were significant differences in intraoperative bleeding between autologous blood transfusion and non-transfusion groups.

**Conclusions:**

The key factors affecting the intraoperative blood loss and postoperative drainage volume include operation methods, operation duration, blood-transfusion modes, and usage of anticoagulants. These results should be taken into consideration in the attempt to optimize operation procedure and improve post-operative recovery.

## Background

Lumbar disc herniation (LDH) is a common contributor to low back pain and low extremity radicular syndrome, especially in middle-aged and elderly population, presenting a quandary to spine surgeons worldwide regarding to the most appropriate intervention [1–3]. The therapeutic approach ranges from conservative medical interventional management to surgery [4–6]. Various studies have confirmed the effectiveness of surgery in the initial management of LDH [7]. Nevertheless, the factors influencing intraoperative procedure and prognosis are still not fully understood.

The surgical outcome of patients with LDH can be affected by many factors, such as patient age, gender, autologous blood availability, preoperative hemoglobin level, and the number of spinal decompressed and fused lumbar [8–10]. In addition, other factors may affect the recovery of patients as well, including intraoperative blood loss, operation duration, use of anticoagulants, postoperative drainage volume, immediate drainage, final drainage, and close and open of drainage tubes. It has been reported that intraoperative blood loss and postoperative drainage volume are important for operation and recovery of patients with LDH [11–15]. However, factors that influence intraoperative blood loss and postoperative drainage volume have not been clarified. Moreover, there are no reports on the effects of the methods of using drainage tubes during recovery on the outcome of spine surgery.

Autologous and/or allogeneic blood transfusions are often applied in lumbar spinal surgery [1–3, 16]. The amount of blood transfusion is increased under certain conditions such as increased intraoperative blood loss and prolonged operation. Furthermore, the volumes of blood transfusion and final drainage can also be affected by several other factors, including postoperative drainage volume, immediate drainage, final drainage, and the methods of using drainage tubes (close or open) [4–6].

In this study, the clinical profiles of mid-aged and elderly patients with lumbar disc herniation were analyzed. We investigated various factors that may influence intraoperative blood loss and postoperative drainage volume, including age, gender, and transfusions. The effects of states of drainage tube (open or closed) on final drainage volume were also studied. We believe that the results will help improve knowledge and practice in spine surgery.

## Materials and methods

### Patients

This retrospective study enrolled 183 patients (109 females and 74 males) with lumbar disc herniation who underwent spinal operation in our department from June 2010 to January 2012. The study was approved by the ethical committee of China-Japan Friendship Hospital (Beijing, China).

### Surgical approach

Patients were in the knee-chest or prone positions; all the procedures were conducted under general anesthesia. A midline incision was made to reflect the paraspinous muscles. The interlaminar spaces were made as described previously by McCulloch and Delamarter [4–6]. In almost all cases, medial borders of superior facets were removed in order to have clear views of the related nerve roots. The fragments of disks were removed as described previously by small annular incisions [4–6]. Blood canals were inspected and the foramens probed for bony pathology or residual disk. Nerve roots were decompressed, leaving them freely mobile during operation. The surgical strategies included decompression alone or decompression with fusion.

### Clinical data

Clinical data were collected for all patients, including age, sex, operation duration, intraoperative blood loss, hamoglobin and hamatocrit levels preoperatively and at discharge, autologous blood availability, preoperative hemoglobin rate, spinal level decompressed and fused number, duration of hospital stay, and history of other diseases, especially hematological diseases.

### Drainage and relevant parameters

For all patients, the plasma drainage tube and disposable drainage bag were emptied every 24 h after the measurement of drainage volume. We collected the data on the drainage time during surgery, intraoperative blood loss, postoperative drainage, and final drainage and operation modes. Drainage time was recorded by the time of blood drainage using a drainage tube during operation.

### Data analysis

Patients were divided into different groups according to gender, age, operation modes (one-level and two-level), different transfusions (autologous and allogeneic blood transfusion), close and open of drainage tube, in the presence or absence of anticoagulant, operation time, immediate drainage, and bone graft methods (autogenous bone implantation, autogenous bone and Cage implant, autogenous bone and artificial bone intertransverse posterolateral implantation, autogenous bone and artificial bone implantation and facet joint fusion). Data are presented as mean ± standard deviation (SD). Differences between various groups were analyzed using one-way analysis of variance (ANOVA) or *χ*^2^–test with SPSS 13.0 software. A *P* value < 0.05 was considered statistically significant.

## Results

### Clinical characteristics

The mean age of the patients was 56.6 and the mean operation duration was 161 min. 83 patients received one-level operations, and 100 patients underwent two-level operations. The mean intraoperative bleeding volume was 477 ml, and the mean volume of intraoperative autotransfusion was 163 ml. The mean immediate drainage volume was 56 ml with a mean drainage time of 2683 s, and a mean final drainage time of 244 s. The mean platelet count in all patients before surgery was 207 × 10^9^/L.

### Factors affecting intraoperative blood loss and postoperative drainage

Intraoperative blood loss is commonly used as a marker and predictor of operation and outcome for patients [4–6]. It has been suggested that various factors including gender, age, operation methods (one-level or two-level operations), and other factors may affect intraoperative blood loss and postoperative drainage in patients with lumbar surgery. The following major factors were analyzed in this study.

- Gender and Age
  There was no statistical difference in bleeding quantity (*P* = 0.079), immediate drainage (*P* = 0.478), and final drainage (*P* = 0.521) during spinal operation between male and female patients (Tables 1 and 2). However, statistically significant differences were observed for intraoperative bleeding quantity (*P* = 0.0014) and final drainage volume (*P* < 0.05), but not for immediate drainage volume (*P* > 0.05) between middle-aged group (<60 years old) and elderly group (>60 years old). There were no sex predominance in mean blood loss volume, and the percentages of the estimated blood volume (EBV) were approximately 6–7 %, consistent with the data previously reported [4–6].

**Table 1:** Effect of gender on intraoperative bleeding, and immediate and final drainage

Patients	Men (*n* = 74)	Women (*n* = 109)	*P*
Intraoperative bleeding (ml)	539.19 ± 424.21	437.06 ± 312.71	0.079
Immediate drainage (ml)	60.61 ± 68.39	53.67 ± 62.33	0.478
Final drainage (ml)	262.91 ± 273.56	239.86 ± 210.09	0.521

**Table 2:** Effect of gender on intraoperative bleeding, and immediate and final drainage

Patients	Men (*n* = 74)		*P* value	Women (*n* = 109)		*P* value
	One-level operation (*n* = 37)	Two-level operation (*n* = 37)		One-level operation (*n* = 46)	Two-level operation (*n* = 63)	
Intraoperative bleeding (ml)	404.05 ± 397.10	674.32 ± 411.91	0.005	341.52 ± 138.23	506.83 ± 380.47	0.002*
Immediate drainage (ml)	56.89 ± 71.54	64.32 ± 65.85	0.643	41.52 ± 36.15	62.54 ± 75.03	0.056
Final drainage (ml)	198.78 ± 157.44	327.03 ± 344.25	0.043	176.30 ± 115.99	286.27 ± 248.87	0.003*

*P value <0.05
- Operative procedure
  There were statistically significant differences in bleeding quantity and final drainage volume between one-level (male patients, *P* = 0.005; female patients, *P* = 0.005) and two-level operations (male patients, *P* = 0.043; female patients, *P* = 0.003) (Table 3). These findings suggested that operative procedures play an important role in intraoperative blood loss and postoperative drainage. However, there was no remarkable difference in immediate drainage volume between one-level and two-level operations in male patients (*P* = 0.643) or female patients (*P* = 0.056) (Table 3). Significant difference in intraoperative bleeding (*P* < 0.001) and final drainage (*P* = 0.001) between one-level and two-level operations, but not in immediate drainage (*P* = 0.115), Correlations were observed in autologous blood transfusion between one-level and two-level operations (Pearson Chi-Square = 4.490, *P* = 0.034) (Table 4).

**Table 3:** Effect of operation levels on intraoperative bleeding, immediate and final drainage in different groups with different ages

Subjects	Ages > = 60 (*n* = 72)		*t* value	*P* value	Ages < 60 (*n* = 111)		*t* value	*P* value
	One-level operation (*n* = 29)	Two–level operation (*n* = 43)			One-level operation (*n* = 54)	Two-level operation (*n* = 57)		
Intraoperative bleeding (ml)	350.00 ± 194.11	518.60 ± 262.77	2.952	0.004	379.81 ± 323.46	606.67 ± 475.22	2.593	0.004*
Immediate drainage (ml)	44.31 ± 62.36	62.79 ± 62.04	1.237	0.220	50.56 ± 51.08	63.51 ± 78.32	1.026	0.307
Final drainage (ml)	173.97 ± 121.07	319.77 ± 328.37	2.284	0.025	192.96 ± 143.47	287.46 ± 253.22	2.435	0.017*

*P value <0.05

**Table 4:** Relationship between operation segments on intraoperative bleeding

Items	One-level operation (*n* = 83)	Two-level operation (*n* = 100)	Pearson Chi-Square	*P* value
Intraoperative autologous blood transfusion	52 (62.7 %)	77 (77.0 %)	4.490	0.034*
Injection of allogeneic blood transfusion	13 (15.7 %)	27 (27.0 %)	3.413	0.065

*P value <0.05
  Statistically significant correlations were noted between intraoperative transfusion quantity and operative modes (one-level or two-level) (Pearson Chi-Square = 10.728, *P* = 0.001; Spearman Correlation = 0.242, *P* = 0.001). However, no relationship was present between transfusion modes and operation modes (one-level and two-level) (Pearson Chi-Square = 2.136, *P* = 0.144; Spearman Correlation = 0.108, *P* = 0.145). Statistical differences were present for intraoperative autologous blood transfusion (Pearson Chi-Square = 4.490, *P* = 0.034 < 0.05), but not for allogeneic blood transfusion (Pearson Chi-Square = 3.413, *P* = 0.065) between patients with different transfusions (Tables 3 and 4).
  The final drainage volume was closely associated with one-level and two-level operations performed independently from other factors (Tables 1 and 2). Furthermore, statistical differences (*P* = 0.001) were shown between groups of one-level operation and two-level operations with respect to final drainage. In addition, regression analysis showed there were significant relationships between final drainage and operation methods (R^2^ = 0.055).
- Blood transfusion
  A correlation was shown between one-level and two-level operations for transfusion modes that included non-transfusion, auto-transfusion, and allogeneic blood transfusion (Spearman’s *r* = 0.237, *P* = 0.001); there were statistical differences (Chi-Square = 11.940, *P* = 0.008) between patients with different transfusions. There were also significant differences in blood loss between autologous blood transfusion for non-transfusion groups (*P* < 0.001) and between allogeneic blood transfusion and non-transfusion groups (*P* = 0.004). However, there was no difference (*P* > 0.05) between these groups for immediate drainage and final drainage (Table 5).

**Table 5:** Relationship between transfusion and intraoperative bleeding

Items	No transfusion	Autoblood	Allogeneic blood	Two kinds of way of transfusion	*X* ^2^	*P* value
	*n* = 34	*n* = 109	*n* = 20	*n* = 20		
Ages	53.32 ± 9.00	57.08 ± 12.12	58.75 ± 12.52	57.60 ± 7.80	4.100	0.251
Immediate drainage (ml)	50.88 ± 48.95	53.85 ± 57.70	94.25 ± 118.49	42.5 ± 35.08	2.227	0.527
Final drainage (ml)	219.41 ± 145.19	230.55 ± 233.22	317.75 ± 210.52	332.75 ± 365.62	7.610	0.055
- Operation duration
  A correlation was noted between operation duration and bleeding quantity (spearman *r* = 0.564, *P* < 0.001) (Fig. 1), which was consistent with previous studies [1–3]. No correlations were observed between operation duration and the following parameters: different bone graft (Chi-square = 2.165, *P* = 0.539), transfusion of allogeneic blood (Spearman *r* = 0.012, *P* = 0.873), immediate drainage (Chi-square = 2.165, *P* = 0.539), and different bone graft (Spearman *r* = 0.047, *P* = 0.530). Correlation analysis demonstrated there were statistical differences between bleeding quantity and operation duration (138.82 ± 44.71 vs. 178.45 ± 58.08, t’ = 5.212, *P* < 0.001) during one-level and two-level operations, and there were also significant differences between operation duration and blood transfusion quantity (166.39 ± 57.06 vs. 134.56 ± 42.34, t = 3.063, *P* = 0.003) (Fig. 1). These findings indicate that operation duration is closely associated with intraoperative bleeding. No correlation was observed between operation duration and transfusion methods (171.50 ± 67.08 vs. 169.12 ± 54.48, t = 0.934, *P* = 0.352) for all patients. Moreover, there are significant correlations between final drainage volume and the following factors: bleeding quantity (*P* = 0.043), transfusion quantity (*P* = 0.032), and immediate drainage volume (*P* < 0.001) (Fig. 2).

**Fig. 1:**
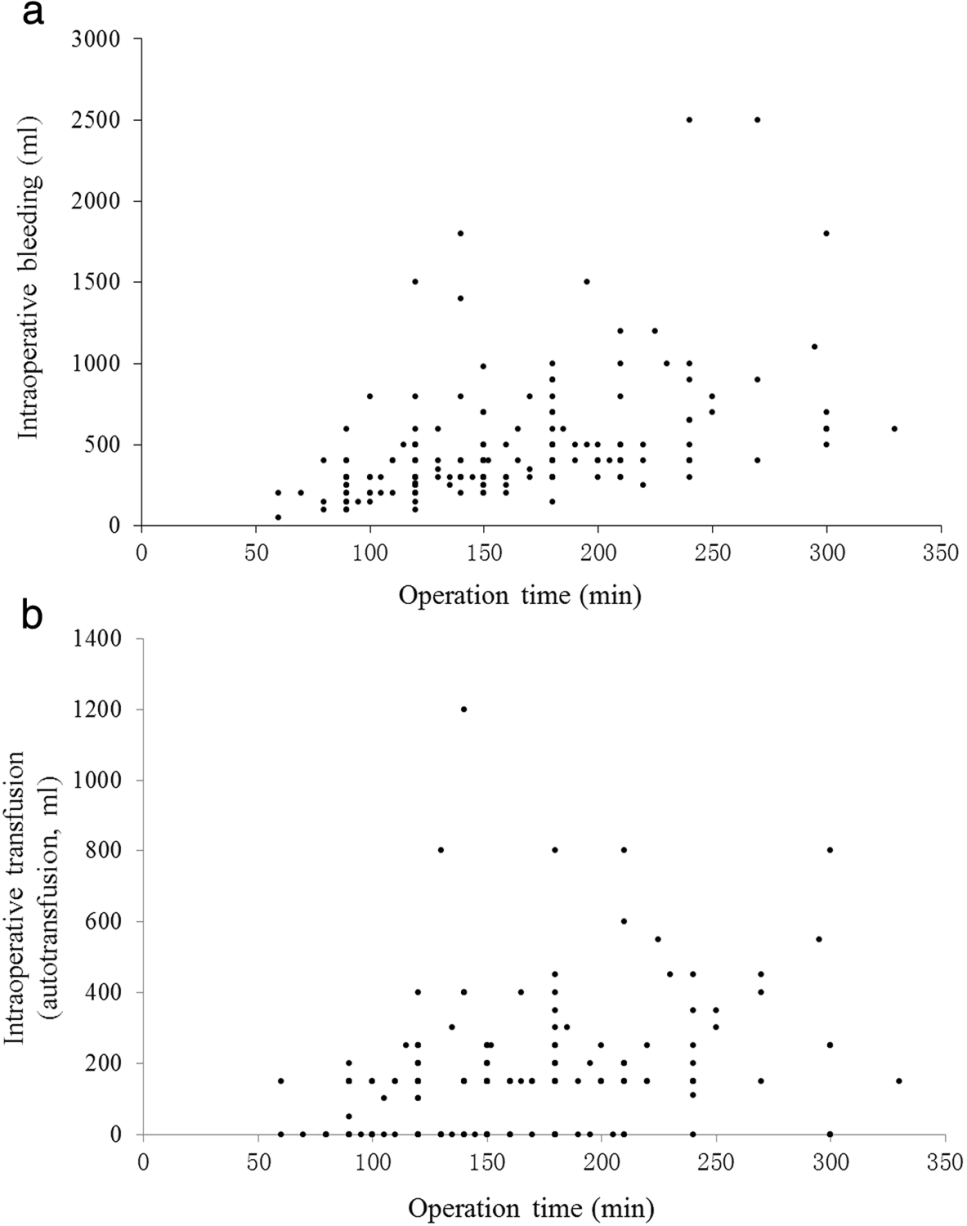
Correlation between operation time and intraoperative bleeding/blood transfusion quantity

**Fig. 2:**
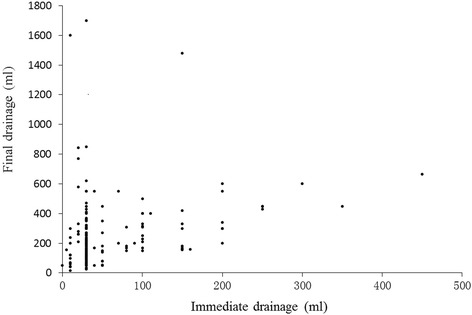
Correlation between immediate drainage and final drainage
- States of drainage tube
  There was no statistical difference between states of drainage states (open and close) and final drainage volume (*P* = 0.280), nor the coagulation index before operation, intraoperative bleeding quantity or postoperative drainage quantity (*P* > 0.05) (data not shown).
  Correlation analysis showed that states of drainage tube were not involved in final drainage volume (*P* = 0.280), and there was no correlation between immediate symptoms of lower limbs and states of drainage tubes (Pearson Chi-Square = 0.350, *P* = 0.554; Spearman Correlation = 0.044, *P* = 0.556) (Fig. 3).

**Fig. 3:**
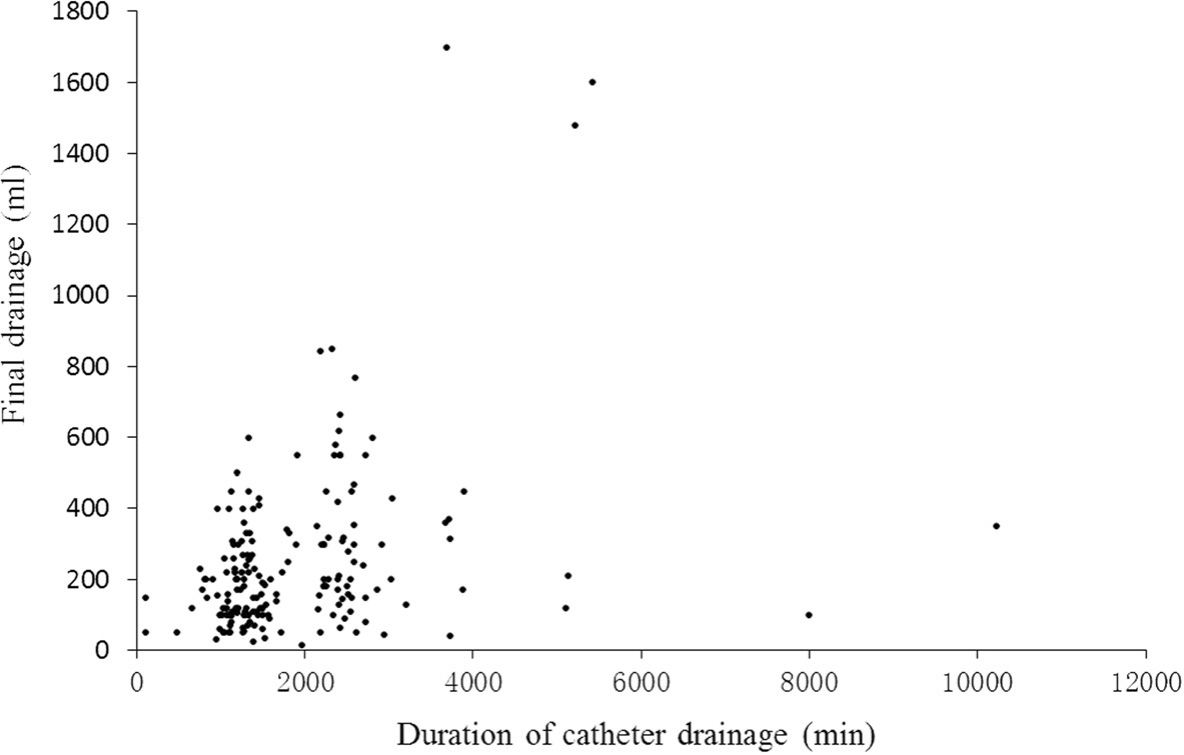
Correlation between duration of catheter drainage and final drainage
- Use of anticoagulants
  No correlation was shown between anticoagulant (antifibrinolytics) use and bleeding quantity during operation (*P* = 0.884, *P* = 0.939), but a correlation was observed between anticoagulant use and drainage quantity after operation (*P* = 0.001).
- Bone graft methods
  No correlation was shown between methods of bone graft and intraoperative blood loss (*P* = 0.879), immediate drainage (*P* = 0.352), or final drainage (*P* = 0.702). In addition, no correlation was shown between methods of bone graft and operation time (*P* = 0.480) (Table 6).

**Table 6:** Relationships between transfusion and types of bone graft

Types of bone graft^a^ and operation modes		Types of blood transfusion				Total
		No transfusion	Autoblood	Allogeneic blood	Both	
Type 1	One-level	14	11	3	1	29
	Two-level	6	23	5	5	39
	Total	20	34	8	6	68
Type 2	One-level	6	29	3	4	42
	Two-level	3	35	7	8	53
	Total	9	64	10	12	95
Type 3	One-level	0	3	1	0	4
	Two-level	1	1	0	0	2
	Total	1	4	1	0	6
Type 4	One-level	4	3	0	1	8
	Two-level	0	4	1	1	6
	Total	4	7	1	2	14

^a^Type 1: Autogenous bone implantation; Type 2: Autogenous bone implantation and Cage implantation; Type 3: Autogenous bone implantation and artificial bone intertransverse posterolateral implantation; and Type 4: Autogenous bone implantation and artificial bone implantation
- Autologous blood transfusion
  A significant difference (t = 2.397, *P* = 0.018) was shown in bleeding during operation between groups with or without autologous blood transfusion. However, there were no differences in immediate or final drainage volume between these groups (Table 5).
- Use of hemostatic agents during operation
  A correlation was shown between the use of hemostatic drugs and bleeding quantity during operation (*P* = 0.036). However, hemostatic drugs (Antifibrinolytics) showed no obvious effect on immediate or final drainage volume (Z = −1.504, *P* = 0.133; Z = −0.494, *P* = 0.621). Furthermore, the use of hemostatic drugs after operation showed no effect on immediate and final drainage (Z = −0.798, *P* = 0.425; Z = −0.676, *P* = 0.499).
- Duration of catheter drainage.
  The duration of drainage tube was positively correlated with the final drainage volume (Spearman’s *r* = 0.333, *P* < 0.001) (Fig. 3).
- The length of hospital stay and recovery in various spinal operation patients
  The length of hospital stay is a common criteria used in the evaluation of operation and recovery of patients 18–20]. The median length of hospital stay for all patients was 16 days and there was no apparent difference in the length of hospital stay between the male and female patients, or between the groups with closed and open drainage tubes, between groups with different operation methods, operation duration, autologous and allogeneic blood transfusion, and use of anticoagulant (all *P* > 0.05).

## Discussion

Previous studies suggest that the recovery of patients with spinal surgery can be affected by various factors that include body weight, gender, preoperative hemoglobin, fusion levels of lumbar spine, bone grafting, usage of anticoagulants, auto-transfusion, allogeneic blood transfusion, and one-level and two-level operations [4–6, 21–24]. However, the effects of intraoperative blood loss, postoperative drainage volume, mode of drainage, and final drainage on patient operation and recovery are poorly understood and require further investigation. In particular, factors determining final drainage need to be investigated. These issues were addressed in this study.

This study provided a comprehensive analysis of factors influencing the operation and recovery of spine surgery. Data demonstrated that operation methods, operation time, autologous and allogeneic blood transfusion and use of anticoagulant affect intraoperative blood loss and postoperative drainage volume. Additionally, the duration of catheter drainage of drainage tube, transfusion, immediate drainage, operation mode, and use of anticoagulant affected final drainage [21–24]. It is worth noting that the mode of drainage (i.e., closed or open) showed no apparent effect on immediate drainage and final drainage volumes.

There were several novel findings in the present study. First, the intraoperative blood loss, postoperative drainage volume, and final drainage are important factors for surgery in spinal patients with lumbar disc herniation. Second, the duration of catheter drainage, autologous blood transfusion, and immediate drainage, one-level and two-level operations, usage of anticoagulant were important for final drainage. Third, closed and open of drainage tube, and states of drainage tubes affected intraoperative blood loss and postoperative drainage volume. Finally, the intraoperative autologous blood transfusion influenced intraoperative bleeding and final drainage.

As the number of patients with LDH continues to increase, spine operation has been far more common [1–3]. Intraoperative blood loss usually serves as marker and predictor of operation and outcome for patients [4–6]. In this study we analyzed various factors, including gender and age, different operative stage (one-level or two-level operations) as well as other factors that may affect intraoperative blood loss and postoperative drainage in patients with lumbar operation. No statistical difference was shown in bleeding quantity, immediate drainage and final drainage between males and females. These findings are contradictory to the results reported in other studies [10]. The reasons for this observation are not clear, but may be related to sample size (there were limited cases in this study) and different patient groups (i.e., age of patients).

The effects of intraoperative bleeding, transfusion and immediate drainage on final drainage were also analyzed using regression analysis. The results demonstrated that bleeding quantity, transfusion, and immediate drainage affected the final drainage. There was no report on the relationship between the mode of drainage (closed or open) and the final drainage. The results showed that states of drainage tube did not affect the final drainage. It is suggested that future studies focus on additional factors that may be important for intraoperative blood loss and postoperative drainage. In addition, the effects of intraoperative blood loss and postoperative drainage on successful spinal operation and patient recovery should be further investigated.

The factors identified as contributors to total blood loss and final drainage volumes in the present study included operation methods, operation time, autologous and allogeneic transfusion and use of anticoagulants. These findings indicate that blood loss and final drainage can be controlled by the surgeon. There was a significant clinical impact on blood loss by using anticoagulants (Antifibrinolytics).

A drainage system was used in spine surgery in the present investigation. Because the drainage was always closed, suction or no suction was the only choice. In addition, clinical practice between China and Western countries has remarkable differences [1–8]. For example, the duration of hospital stay is often much shorter in Western world than that in China.

There were four fusions per levels, 46 decompressions and 32 herniated discs in the present study. Although one is quite surprised to find herniated discs in a study on transfusion, transfusion indeed plays important roles in surgery of patients with herniated discs, just as shown in the present study. It is obvious that the data showed that there were differences in blood loss between autologous transfusion groups and non transfusion groups. Indeed, it was not required an autologous donation for single level fusion or herniated disc surgery in the present study.

## Conclusions

In conclusion, intraoperative blood loss and postoperative drainage volume are important for spinal patient recovery, and are affected by various factors. A better understanding of these factors would greatly improve the patient care and outcome of spine surgery.

### Limitations

There are several limitations to our study. Our results were analyzed in a retrospective fashion, and the cohort size was relatively small. The follow-up data were not available; thus the related long-term prognosis still needs further studies.
